# The management of opiate use disorders in France: results of an observational survey of general practitioners

**DOI:** 10.1186/s13722-015-0037-2

**Published:** 2015-06-28

**Authors:** Pierre Poloméni, Claude Bronner, Fréderic Fry, Bruno Ravoninjatovo, Mélina Fatseas

**Affiliations:** Hôpitaux Universitaires Paris Seine St Denis, Site René Muret, Avenue du Dr Schaeffner, Sevran, 93270 France; 2 rue de Haslach, 67200 Strasbourg, France; CSAPA Dune, Parvis de la préfecture, Immeuble les Oreades, 95000 Cergy, France; 151 Bis rue de Courcelles, 51100 Reims, France; Laboratoire de psychiatrie, Département d’addictologie, Centre hospitalier Charles Perrens, 146 bis, rue Léo Saignat, Bordeaux, 33076 France

**Keywords:** General practitioners, Specialists, Substance use disorders, Opiates, Heroin, Opioid-agonist treatment, Opioid-agonist management

## Abstract

**Background:**

When opioid-agonist treatments were approved in France in 1995, opiate use disorders began to be managed and treated by general practitioners (GPs), who have since then been encouraged to treat substance use disorders (SUDs) for heroin and other illegal substances. The objective of this study was to describe rates of: 1) SUDs in general practices in France; 2) characteristics of GPs treating SUDs; and 3) clinical practices surrounding SUDs. To place these data in the context of SUD treatment, we also gathered information from practicing SUD specialists.

**Methods:**

Between December 2011 and January 2012, a nationally representative sample of GPs and SUD specialists were interviewed by phone, using a 12-item questionnaire that covered number of SUD patients, types of SUDs, and treatments. Data collected were confidential, and analysis was blinded with regard to physician identity.

**Results:**

Forty-four percent of GPs and 68 % of specialists were included in the analysis. The mean number of patients estimated to have been seen at least once in the previous year was 3036 for GPs and 920 for specialists. Ninety-six percent of GPs reported having patients with SUDs. Tobacco, alcohol, and psychoactive drugs were the SUDs most frequently encountered by GPs, whereas tobacco, alcohol, heroin, and cannabis were most frequently encountered by specialists. Forty-three percent of GPs saw at least one patient with a heroin use disorder (HUD), and 82 % of GPs treating patients with HUDs had prescribed an opioid-agonist treatment during the previous 12 months.

**Conclusions:**

The results of this study suggest that a large number of GPs now treat patients with opiate use disorders and that doctors appear to be convinced of the benefits of opioid-agonist therapy and have overcome their initial concerns. This represents a significant change in practice patterns since the introduction of opioid-agonist treatments in France.

## Background

In 1995, opioid-agonist therapy (OAT) was approved in France as maintenance therapy for major opiate substance use disorders (SUDs) [1], with the stipulation that initiation of methadone treatment take place in specialized SUD centers and that general practitioners (GPs) only be allowed to renew methadone prescriptions and to prescribe buprenorphine-based treatments alone or in combination with naloxone. At the end of the 1990s, the treatment and management of SUDs was reorganized with the creation of specialized centers (Centres d’Accompagnement et de Prévention en Addictologie [CSAPA]), which had broader mission statements, centers for the reduction of risk (Centres d’Accueil et d’Accompagnement à la Réduction de risques pour Usagers de Drogue), and an SUD subspecialty in medical curriculums [2]. As a result of these measures, GPs, who were encouraged to be more involved in the treatment of opiate use disorders, have become key players in the treatment of SUDs in France. Data published in 2012 showed that 260,000 patients were being treated with agonist strategies, most of which were being prescribed by GPs in private practice [3]. In addition, GPs have been able to take a broader approach and integrate the management of opiate use disorders with the management of other SUDs for alcohol, tobacco, and other prescription medications.

Our study describes current treatment practices of GPs in France. Data describing the prevalence of SUDs in general practices, the role of GPs in the management of opiate use disorders, and their prescription choices were gathered through a phone survey. To place these data in the context of SUD treatment in France, we also gathered information from practicing SUD specialists.

## Methods

This pilot study, conducted by the healthcare department of the French Institute of Public Opinion (Institut français d’opinion publique), was a quantitative survey of GPs and specialists affiliated with CSAPA centers. Health professionals were interviewed by phone using contact information obtained from a national roster. All physicians provided explicit informed consent, and all data were treated confidentially. Data collection and analysis were performed according to the requirements put forth by the National Commission for Data Protection and Liberties. The survey was conducted from December 12, 2011, to January 6, 2012.

### Questionnaire

The questionnaire included 12 questions designed to assess current practices in the treatment and management of opiate use disorders. The survey started with screening and demographic questions designed to collect information about provider specialty (GP, GP specialized in SUDs, specialist [psychiatrist or other]); main place of practice (private practice, specialized SUD center, other); percentage of time spent practicing medicine, teaching, or doing administrative tasks; number of years practicing medicine; geographic location of practice; and physician age.

For the five SUD-specific questions, physicians were asked to estimate the number of patients in their active file (with active file being defined as the total number of patients seen at least once in the last 12 months) and to base their responses on these patients. Questions were designed to collect data about whether physicians were involved in the management of patients with SUDs; the number of patients suffering from an SUD for alcohol, tobacco, cannabis, heroin, stimulants (amphetamines, cocaine, crack, ecstasy, etc.), hallucinogens (LSD, mushrooms, datura, etc.), analgesics (codeine, oxycodone, tramadol, etc.), psychoactive drugs (benzodiazepines, etc.), OAT not prescribed by a physician, and non-SUDs (games, gambling, shopping on credit, sex, Internet); the number of patients with a heroin use disorder (HUD) who were also dependent on other substances (alcohol, tobacco, ecstasy); the number of patients suffering from opiate use disorders who were prescribed an OAT (in general); and lastly, the number of patients suffering from opiate use disorders who were prescribed methadone, buprenorphine (Subutex® or generics), or another OAT (breakdown per treatment).

### Data handling and analysis

To obtain a nationally representative cohort of GPs, quotas for sex, age, and geographic region were defined using 2011 medical population data from the international database of the Cegedim Group (Boulogne-Billancourt France), which described 60,000 GPs. To obtain a nationally representative cohort of specialists, quotas for gender and geographic region were also established based on 2011 data from the Cegedim Group. Within these quotas, GPs and SUD specialists were selected randomly by a computer-generated program. Physician sample sizes were determined, taking into account cost of study and reliability of responses. For a cohort of 450 GPs, the 95 % confidence interval for a response rate of 30 % was estimated at 25.8–34.2 %.

To maintain physician anonymity during data analysis, names of physicians were replaced by a computer-generated random code. Data for GPs and specialists were analyzed using descriptive statistics. Quantitative variables were reported as means ± standard deviation, and qualitative data were reported as *n* (%). When totals for demographic data and type of SUD were calculated, the data from the GPs and specialists were weighted to match the current distribution of physicians in France. As this study is primarily a description of GPs, comparisons were only performed for general practice and demographic questions. Comparisons between means were made using Student’s T-tests. Percentages were compared by chi-square tests between pairs. Significant differences were defined at a confidence level of 95 %. Data were analyzed using software from the Centre for Open Software Innovation.

## Results

The response rate was 44 % for GPs and 68 % for SUD specialists. Data from 450 GPs and 63 specialists were included in the analysis.

The cohort of GPs was 71 % male. Mean age was 52 years, and the mean number of years in practice was 22. Fifty-six percent of GPs had been practicing for at least 21 years (Table 1). The cohort of specialists was 54 % male. Mean age was 52 years, and the mean number of years in practice was 15. Twenty-two percent of specialists had been practicing for at least 21 years. GPs reported seeing a mean of 3036 patients at least once a year. When weighted totals were calculated, the mean age of physicians treating SUDs in France was 52 years, and the mean number of years in practice was 21. Differences between the specialist group and the total group were statistically significant for gender and mean number of years in practice. Specialists reported seeing a mean of 920 patients at least once a year.

**Table 1:** Socio-demographic characteristics: physicians

	Total	General practitioners	SUD specialists
	(N = 513)	(n = 450)	(n = 63)
Gender, % male	71 %	71 %	54 %*
Age			
Mean ± SD, years	52 ± 10	52 ± 10	52 ± 9
< 46 years, %	23 %	23 %	23 %
46–50 years, %	13 %	13 %	14 %
51–55 years, %	20 %	20 %	31 %
≥ 56 years, %	44 %	44 %	32 %
Number of years of practice			
Mean ± SD, years	21 ± 11.1	22 ± 11	15 ± 10*
0–5 years	12 %	12 %	22 %
6–10 years	10 %	10 %	22 %
11–15 years	7 %	7 %	14 %
16–20 years	15 %	15 %	19 %
21–30 years	33 %	33 %	15 %*
≥ 31 years	22 %	23 %	7 %*
Regional location			
Ile-de-France^a^	16 %	16 %	22 %
North-East	23 %	23 %	23 %
North-West	22 %	22 %	28 %
South-West	13 %	13 %	10 %
South-East	26 %	26 %	17 %

*SD* standard deviation, *SUD* substance use disorder

^a^Paris and surrounding areas

*p < 0.05 vs. the total population

### Patients with SUDs seen by GPs and specialists

**Fig. 1:**
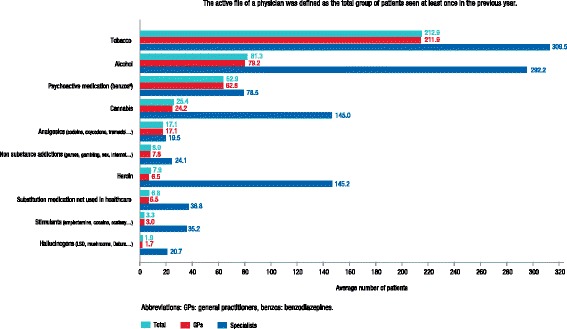
Types of substance and non-SUDs in active files of physiciansData were collected about whether physicians were involved in the management of SUDs and non-SUDs. The active file of a physician was defined as the total group of patients seen at least once in the previous year. Abbreviations: GPs = general practitioners, benzos = benzodiazepines

Ninety-six percent of GPs (n = 433) reported having at least one patient with an SUD. The mean number of patients that these GPs reported seeing at least once a year was 3061. Tobacco, alcohol, and psychoactive drugs were the most frequently reported SUDs being managed, with a mean of 212 (median of 77), 79 (median of 17), and 63 (median of 14) patients per GP, respectively (Fig. 1). General practitioners also reported seeing patients with an analgesic substance use disorder (mean of 17; median of 2 patients per GP), a HUD (mean of 7; median of 0 patients per GP), and nonprescription OAT use disorders (mean of 7; median of 0 patients per GP).

Ninety-eight percent of specialists (n = 62) reported having patients with SUDs. The mean number of patients that these specialists reported seeing at least once a year was 936. Tobacco, alcohol, heroin, and cannabis were the most frequently reported SUDs being managed, with a mean of 310 (median of 144), 292 (median of 198), 145 (median of 28), and 145 (median of 68) patients per specialist, respectively (Fig. 1). Specialists also reported seeing patients with analgesic use disorders (mean of 19; median of 4 patients per specialist) and nonprescription OAT use disorders (mean of 37; median of 9 patients per specialist).

### Opioid-agonist therapy

Of the 450 physicians surveyed, 35 % of GPs prescribed an OAT to an average of 4.2 patients (median of 0). Of these patients, 59 % received a prescription for buprenorphine, and 37 % for methadone. In CSAPA centers, buprenorphine accounted for 37 % of OAT prescriptions, and methadone for 63 %.

### Heroin use disorder

Forty-three percent (194/450) of GPs saw at least one patient with an HUD per year. Thirty-six percent of GPs reported having between 1 and 10 HUD patients, while 1 % reported having more than 50 HUD patients. Among GPs who had HUD patients in their practice (n = 194), the mean number of HUD patients was 14 (median of 3). General practitioners reported that 83 % of HUD patients had an SUD for another substance, most often alcohol or tobacco.

Eighty-two percent (52/63) of specialists saw at least one HUD patient per year. Among these specialists (n = 52), the mean number of HUD patients was 174 (median of 40). Specialists reported that 76 % of HUD patients had an SUD for another substance.

**Fig. 2:**
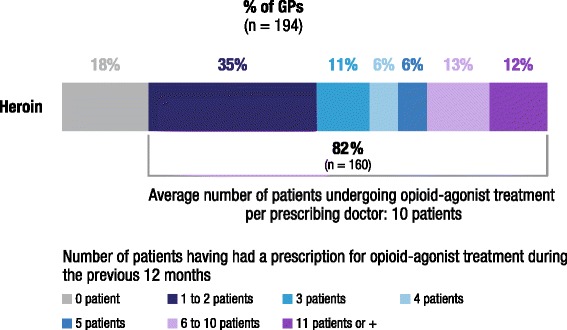
Percent GPs who prescribed opioid-agonist treatment in the previous 12 months

Eighty-two percent (160/194) of GPs seeing HUD patients had prescribed OAT during the previous 12 months. This corresponded to an average of 10 patients per prescriber. Practices, however, were heterogeneous: 35 % of physicians treated only 1 or 2 patients, and 12 % treated 11 patients or more (Fig. 2). Characteristics of GPs prescribing OAT were not significantly different (in age, sex, or region) compared to other physicians in the sample (data not shown).

## Discussion

GPs, which are present throughout the course of patient care, are often the first to witness the emergence of SUDs and are uniquely positioned to identify associated SUDs. This study, which was a phone survey of a nationally representative sample of French GPs, confirmed the fact that GPs have integrated the treatment of SUDs and non-SUDs into their clinical practice. Ninety-six percent of GPs reported having patients with SUDs. In addition, GPs in France are now invested in the care of HUD patients, as 43 % of GPs reported treating HUDs and 82 % of these GPs reported prescribing OATs. GPs also reported that 83 % of HUD patients had an associated SUD. Lastly, GPs also reported having a significant number of patients with opiate analgesic use disorders, an area of SUDs that has not been studied extensively in France.

The first prevalence estimates of problem drug use in France were made in the mid-1990s. In 1995, the number of persons with HUDs was estimated to be at least 160,000, using a demographic method [4]. In 1999, the number of problem opiate or cocaine users was estimated between 146,000 and 180,000 [4]. In 2006, the estimate jumped to 230,000 users [4]. In 2012, the French Monitoring Center for Drugs and Drug Addiction (OFDT) estimated the number of problem drug users (heroin, cocaine, or amphetamines) to be 260,000, of whom 81,000 were using the intravenous route [5]. Although these data suggest a significant increase in the number of problem SUDs since 2006 [4], this impression is misleading [5] as the methods and study populations have changed. The definition of the group of interest, for example, has progressed from heroin users to problem opiate users (1995), to problem opiate or cocaine users (1999), and finally, to intravenous drug users or regular users of opiates, cocaine, or amphetamines (2006).

Distribution of GPs in the different regions and the characteristics of the physicians in our study are similar to those previously documented in the Cegedim Group registry. For example, the Cegedim Group reports that 73 % of GPs in France are male; in our sample 71 % of GPs were male. This comparison suggests that the algorithm that was used to select a nationally representative cohort of GPs was successful and that our data can be extrapolated to all of France, with little risk of introducing significant bias.

When we extrapolated our numbers to all French doctors, we found that there were an estimated 360,000 people suffering from opiate use disorder in France. This incidence of opiate use disorder is higher than expected. When a similar extrapolation was made with data from the “General Practitioners’ Health Barometer 2009” study, which surveyed 2083 physicians in France and reported similar socio-demographic profiles [6], the number of people suffering from HUD in France was estimated to be 110,000. This difference may in part be due to the fact that our survey did not make the distinction between experimentation, regular use, and dependence, as defined by the OFDT. In fact, it is likely that the estimated number of persons with an HUD described by the GPs in this study (500,000 people according to the OFDT database) included some experimental users of heroin [3]. Furthermore, these estimates should be interpreted with caution, as there is also the possibility that patients consulted several doctors. Medical nomadism, though in decline, has been confirmed by several studies. In 2007, patients saw on average two GPs per year, and 25 % of patients saw at least three physicians per year [7]. In the Loire region, 14 % of patients have been reported to have consulted multiple doctors [8].

Our data support previous data that show that the changes put in place at the end of the 1990s have increased the involvement of GPs in the treatment of SUDs. Since the introduction of high-dose buprenorphine (HDB) in private practice, a survey of 1186 GPs showed that while 53 % of doctors had seen an HUD patient in the previous year, only 24 % were involved in ongoing care, and only 13 % of doctors said that when asked for OAT, they would agree to prescribe buprenorphine [9]. In the 2009 “General Practitioners’ Health Barometer” study, about 50 % of GPs reported seeing at least one patient with an opiate use disorder per month, and 87 % of GPs who saw at least one patient with an opiate use disorder per month prescribed OAT [6]. Similarly, in our study, 43 % of GPs reported seeing at least one HUD patient per year, and 82 % of these GPs had prescribed OAT during the previous 12 months. These data are consistent with data that suggest that more than 60 % of patients with an opiate use disorder are treated [10] and that 70 % of heroin users received OAT [11].

In addition, our study shows that 59 % of prescriptions written by GPs for OAT were for buprenorphine and that 37 % were for methadone. These data suggest a shift compared to 2007 data, when HDB prescriptions accounted for 80 % of nonhospital prescriptions for agonisttherapies, and compared to 2010 data, which showed that HDB accounted for nearly 75 % of all nonhospital prescriptions [7, 12]. Data from the French national health insurance service support our findings, as the increase in reimbursement requests rose dramatically between 2004 and 2010 for methadone (+276 %) compared to HDB (+29.3 %) [12].

Better physician education about the pathology, treatment strategies, and modalities of OATs [13] are likely to have contributed to this positive progression in treatment practices. Fears about overdoses associated with OAT prescriptions [14] appear to be dissipating, possibly because they are offset by the fact that problems with aggression tend to decrease with OAT. In addition, the fact that patients suffering from SUDs consider the relationship with their physician as an important factor may also have shifted patients towards seeking help from their GPs who, by definition, provide a more rounded approach to patient care [1, 15]. However, the realities of clinical practice often impose time constraints, which limit physician availability, and various studies have shown the necessity of remaining cautious due to the risk of overdose, especially in combination with benzodiazepines [16] and alcohol [17].

Among physicians who do treat patients with an HUD or an opiate use disorder, the distribution of patients is uneven [8, 18]. Our data show that GPs can be divided into two broad groups: one group that sees a few, clearly identified, and regular patients, and one group that sees many users. We show that 36 % of GPs treat 10 or fewer HUD patients, whereas 2 % of GPs treat 30 or more HUD patients per year. These data are consistent with previously published data which have shown, for instance, that in the Loire region, a single doctor treated 112 patients with an opiate use disorder using OAT, whereas 127 doctors in the same region treated only 1 patient per practice [8]. In addition, a 2006 study showed that 26 % of prescribing doctors in France treat 75 % of all patients [10]. Better distribution of patients could alleviate some of the time burdens and generally improve care for SUDs.

This study found few differences in physician practices across regions (data not shown). These data differ from those reported by other studies, which have shown heterogeneity in OAT prescription practices among GPs [19, 20]. In the large OFDT study of the 13 Primary Sickness Insurance Funds, for example, 35 % of GPs prescribed OAT during the first half of 2002; but in Metz and Lille, the proportion was larger (50 %) [20]. A more recent study in the Nord-Pas-de-Calais region showed that GPs prescribed almost all the OAT for that region (98.2 % in 2009).

### International context

France is not the only country in which GPs have been heavily implicated in the treatment of SUDs. In the United Kingdom, GPs play a pivotal role in managing opiate use disorder [21]. Three times more doctors were faced with opiate users in 2001 compared to 1985, and 50 % of them prescribed OAT and, more specifically, methadone. However, questions have been raised about the quality of care, as the prescribed dose of methadone (around 30 mg), even if it is relatively safe, is not sufficient for effective “maintenance” [21]. It has been suggested that HDB, which was prescribed by fewer than 5 % of British doctors, could provide a better risk/benefit ratio [21]. By contrast, in Australia and in Germany, the focus has been on the obstacles associated with OAT and as a result, GPs have not been extensively involved in the treatment of these patients [22–24].

### Study limitations

The data in this study must be interpreted in light of the limitations of its design. Physicians were asked to estimate number of patients over the phone. The level of subjectivity in this method of data collection is high. In addition, the questionnaire is not a validated tool and therefore, the sensitivity and reproducibility of these data cannot be determined. As previously discussed, the questionnaire did not differentiate between modalities of use or degree of dependency, and therefore did not address notions of recreational use versus SUD. In addition, our study did not evaluate outcomes of OAT administered by GPs, and therefore we cannot comment on whether this shift in practices has led to an improvement in care. Lastly, it is important to note that the number of SUDs reflect the involvement of physicians. For example, the number of tobacco use disorders reflects number of patients managed by physicians, and not the number of smokers in France.

## Conclusions

Shifts in policy and the development of new treatment options over the last 20 years have progressively increased the role of French GPs in the treatment of SUDs, and particularly, in the management of opiate use disorder. This study provides new data on the practices of physicians and shows that 96 % of physicians report treating SUDs. Among physicians who treat HUD patients, 82 % prescribe OATs. These data suggest that doctors appear to be convinced of the benefits of OAT and have overcome their initial concerns [25].

Looking forward, we would like to suggest that GPs continue to be encouraged to focus on new therapeutic modalities (drug or nondrug), because prescription OAT, at an appropriate dosage, optimizes efficacy and reduces misuse [26, 27]. The value of OAT in new clinical situations, such as opioid analgesic use disorders, needs to be explored further [28].

## Abbreviations

CSAPA: Centres d’Accompagnement et de Prévention en Addictologie
GP: General practitioner
HDB: High-dose buprenorphine
HUD: Heroin use disorder
OFDT: French Monitoring Center for Drugs and Drug Addiction
OAT: Opioid-agonist therapy
SUD: Substance use disorder
